# Health professions’ students have an alarming prevalence of depressive symptoms: exploration of the associated factors

**DOI:** 10.1186/s12909-016-0794-y

**Published:** 2016-10-21

**Authors:** Eiad AlFaris, Farhana Irfan, Riaz Qureshi, Naghma Naeem, Abdulaziz Alshomrani, Gominda Ponnamperuma, Nada Al Yousufi, Nasr Al Maflehi, Mohammad Al Naami, Amr Jamal, Cees van der Vleuten

**Affiliations:** 1 Department of Family and Community Medicine, College of Medicine, King Saud University, Riyadh, Saudi Arabia; 2 Medical Education Department, Batterjee Medical College, Jeddah, Saudi Arabia; 3 Department of Psychiatry, College of Medicine, Al Imam Mohammad Ibn Saud Islamic University, PO Box 7544, Riyadh, 13317-4233 Saudi Arabia; 4 Medical Education, Medical Education Development and Research Centre, Faculty of Medicine, University of Colombo, Colombo, Sri Lanka; 5 Biostatistical Consultant CDRC, College of Dentistry, King Saud University, Riyadh, Saudi Arabia; 6 General Surgery Division, KKUH, KSU, College of Medicine, King Saud University, Riyadh, Saudi Arabia; 7 College of Medicine, King Saud University, Riyadh, Saudi Arabia; 8 Department of Educational Development and Research, School of Health Professions Education, Faculty of Health, Medicine and Life Sciences, Maastricht University, Maastricht, The Netherlands

**Keywords:** Depression, Health science students, Mental health, Wellness, Prevalence

## Abstract

**Background:**

There is a need to better understand the depression phenomenon and to clarify why some students become depressed and others don’t. The purpose of this study was to compare the prevalence of depressive symptoms among health professions’ (HP) students, and to explore the association between socio-demographic factors (e.g. year of study, discipline, gender) and depressive symptoms.

**Methods:**

In this descriptive–analytic, cross-sectional study, stratified proportionate sampling strategy was used to select the study sample during the academic year 2012–2013. The students from four health professions’ schools situated within a large, public university located in Riyadh, Saudi Arabia were screened for depressive symptoms using the 21-item Beck Depression Inventory (BDI II). Chi-square test, student *t*-test and ANOVA were used to compare different categorical variables.

**Results:**

The overall response rate was 79.0 %, the highest among dental students 86.1 %, and lowest among nursing (49.7 %). The overall prevalence rate of depressive symptoms was 47.0 %; it was highest among dentistry students (51.6 %), followed by medicine (46.2 %), applied medical sciences (AMS) (45.7 %) and lowest among nursing students (44.2 %). A statistically significant association was found between the presence and severity of depressive symptoms on one hand and the female gender (*p* = 0.000) and year of study on the other hand.

**Conclusion:**

This study seems to indicate an alarming rate of depressive symptoms. Female gender, dentistry, the third year for all schools and fifth year for medicine and dentistry have the highest association with depressive symptoms. Future studies may be needed to explore further the reasons and explanations for the variation in the prevalence of depressive symptoms among these groups. The factors that deserve exploration include curricular variables and personal factors such as the students’ study skills.

**Electronic supplementary material:**

The online version of this article (doi:10.1186/s12909-016-0794-y) contains supplementary material, which is available to authorized users.

## Background

Depression is a very common mental health problem, affecting a significant proportion of health professions’ (HP) students [1–3]. It was found that students who suffer from both anxiety and depression are usually at risk of poor academic performance with lower grade point averages [4, 5]. Depression is a multi-dimensional disorder that adversely affects inter-personal, social and occupational spheres of students’ life [6]. Furthermore, the problem is treatable [7] and modifiable, as managing depression has been shown to improve academic performance [5]. Therefore, students’ mental health could be put among the top priorities of universities throughout the world.

Reports have found depressive symptoms among HP students to be higher than age matched populations [3, 4, 8–11]. A high prevalence rate of depressive symptoms among pharmacy students (52.4 %) has been found in a school in the United States [12]. The reported worldwide prevalence of depression among medical students varies ranging from 8 % in multiple US Universities using the Center for Epidemiologic Studies-Depression scale (CES-D) Survey to as high as 70 % in medical schools of Pakistan using the Aga Khan University Anxiety and Depression Scale [13, 14]. A recent meta-analysis that included 77 studies and 62,728 medical students found the global prevalence of depression to be 28.0 %. (95 % confidence interval [CI] 24.2–32.1 %) [15].

Nursing and dental students experience considerable stress and depressive symptoms during their training [16–19]. Dental schools are known to have highly demanding and stressful learning environments [20] and higher levels of depression, obsessive-compulsive disorders, stress-related psychosomatic activity and increased mood disturbances and interpersonal sensitivity than age-matched norms [19, 21–24]. Applied medical sciences (AMS) students have scored higher on loneliness, anxiety and depression than medical students [25].

Many factors can precipitate psychological illnesses among undergraduates, such as adjustment to the novel school environment, information overload, lack of leisure time, financial constraints, family-related stressors and the competition for higher grades [4, 26, 27]. Other factors include poor coping strategies, study skills and motivation [28–30]. With regard to most of these stressors, ‘resilience’ is an attribute that needs to be promoted among students, especially those vulnerable to depressive symptoms [28–30].

A literature search using PubMed, ERIC and Google Scholar found limited studies that compared depressive symptoms amongst students of different HP schools [30–34]. Most of the studies involved students of one discipline. Hence this study aimed to understand the depression phenomenon better through comparing the prevalence of depressive symptoms in different HP schools namely medicine, nursing, dentistry and AMS, and to explore the socio-demographic factors associated with depressive symptoms.

## Methods

### Study design

Cross sectional descriptive study.

### Setting of the study

The study targeted students attending the schools of Medicine, Dentistry, Nursing, and AMS at the King Saud University (KSU) of all academic years and both genders during the academic year 2012–2013. Like other Saudi undergraduate HP schools, KSU operates on a single-gender basis (teaches the two genders separately). The schools’ students are almost all Saudi (a fairly homogenous set of students with similar ethnicity and cultural backgrounds). Students receive a monthly financial reward (267 USD), and they do not pay university fees or tuition. The chance of getting enrolled to the school once they finish secondary school does not differ by gender or marital status.

In KSU, the number of study years is six for medicine and dentistry followed by one-year internship. Once getting the certificate of completing the school requirement and internship, they are eligible to practice or begin their vocational board/post graduate clinical training. This takes four or more years depending on the field before being eligible to a final board exam. The pharmacy school is a five-year program, while the AMS and nursing schools are four-year program followed by one-year internship. The AMS school in KSA has 12 specialties such as radiological science, clinical lab, physiotherapy, optometry, speech therapy and clinical nutrition etc. The jobs for the AMS graduate are either technologist in the hospitals or teaching assistant in universities.

### Sampling

To achieve the study objectives, a sample of 1,500 students was recruited based on the assumption that the prevalence of depression is 30 ± 3 % at 10 % level of significance. The RaoSoft [35], website was used to estimate the required sample size based on the estimated parameters for the study population. Stratified sampling strategy using proportional allocation method was used to determine the number of students based on their school, gender and year of study. Microsoft Excel program was used to randomly select the study sample.

### Data collection

A short demographic questionnaire (six items) was developed to gather information from the participants, namely age, gender, school, marital status and the year of study. The BDI-II was administered at the same time. The data collection lasted around two months. Participation was voluntary.

#### Beck Depression Inventory (BDI-II)

This inventory is a 21-item self-report instrument for measuring the severity of depression [36] with four response options ranging from 0 to 3 for each item. The total maximum score for all items thus is 63 [37]. A score of 0–13 is considered minimal, 14–19 mild, 20–28 moderate, and 29–63 severe depression [36]. The BDI-II is a relevant psychometric instrument with broad applicability for research and clinical practice worldwide [38, 39]. The BDI-II Inventory was selected because of its wide use and its specificity and sensitivity at detecting depression among university students, its high reliability and improved concurrent and content validity based on available psychometric evidence [38].

### Quality assurance

It was decided to conduct the data collection process through training a group from the students themselves. Therefore, trustworthy team leaders (data collectors) were selected based on the recommendations of the vice deans or the school administrators. For each school and study year, team leaders were assigned with the ratio of one leader for each 25–30 students. Team leaders were awarded some financial incentives (500 SR = 133 $) on the condition that they perform their task properly and efficiently.

The 21-item BDI II inventory was administered to the sample in all the health schools of KSU in Riyadh, Saudi Arabia. All team leaders had a training session in which they were inspired to be diligent and honest. They were informed about the aims of the study and the data collection process; anonymity and confidentiality were emphasized. The instrument (demographic and BDI II questionnaire in a printed format) along with a covering letter, highlighting the aims and objectives of the study, were distributed to the students through the students’ team leaders. Students returned the completed questionnaires either immediately or one day later.

### Statistical analysis

BDI-II scores and demographic data were entered into statistical Package for Social Sciences (SPSS version 19). Descriptive statistics along with Chi-square test (after checking the cell count fit), student *t*-test and ANOVA were used to compare the prevalence of depressive symptoms according to the discipline of study and other socio-demographic factors; i.e. gender and year of study. A *p* value of less than 0.05 was considered significant.

## Results

Overall, 1186 students filled out the BDI-II. The overall response rate for both the BDI and the demographic questions was 79.0 % (dental 86.1 %, AMS 79.2 %, medicine 76.9 % and nursing 49.7 %). The highest number of students included in the study was from the school of AMS (39 %), followed by the school of medicine (37 %), and the least proportion was from the school of nursing (9 %) (Table 1). The mean age of the students was 21.34 + 1.58 years.

**Table 1:** Association between level of depressive symptoms and socio-demographic characteristics among students from four HPs Schools

Socio-demographic characteristic	Number (%)	Mean score (SD)	No (%) with depression	SIGNIFICANCE ANOVA df, f value and *P* value
Gender^a^				
Male	667 (56.3)	12.4 (9.3)	263 (39.4)	0.000^a^
Female	518 (43.7)	16.2 (9.1)	291(56.1)	
School				
Medicine	435 (36.7)	14.3 (9.7)	201 (46.2)	3,0.530,0.662
Dentistry	186 (15.7)	14.5 (10.6)	96 (51.6)	
Applied Medical sciences (AMS)	461 (38.9)	13.9 (8.6)	211 (45.7)	
Nursing	104 (8.7)	13.3 (10.0)	46 (44.2)	
Year				
1st	181 (15.4)	14.0 (8.3)	87 (48.1)	4, 2.899,0.021
2nd	249 (21.1)	14.4 (9.3)	110 (44.2)	
3rd	262 (22.2)	15.1 (9.7)	133 (50.7)	
4th	401 (33.8)	13.0 (9.7)	172 (42.9)	
5th^b^	86 (7.3)	15.9 (9.5)	49 (57.0)	

^a^for this variable i.e. gender, the test was student *t* test

^b^5^th^ year is only for college of Medicine and Dentistry

The overall prevalence of depressive symptoms was found to be 47.0 % (Table 2). The prevalence rate of depressive symptoms was the highest among the students of dentistry (51.6 %), followed by medicine (46.2 %), AMS (45.7 %) and was lowest among nursing students (44.2 %) (Table 1). The difference between the schools was statistically insignificant (*χ*2 = 11.847, *p* = 0.222).

**Table 2:** Association between gender and level of depressive symptoms among students by school

School	Gender	Minimal %	Mild %	Moderate %	Severe %	Mean score	Total morbidity %	*P value
Medicine	Male	60.8	21.2	10.4	7.7	12.6	39.3	0.001
	Female	43.4	24.0	20.0	12.6	17.0	56.6	
Dentistry	Male	59.8	17.1	18.8	4.3	11.6	40.2	0.000
	Female	29.0	24.6	27.5	18.8	19.5	70.9	
AMS	Male	57.0	22.1	15.3	5.5	13.4	42.9	0.618
	Female	51.1	25.3	16.4	7.1	14.4	48.8	
Nursing	Male	76.4	7.3	12.7	3.6	9.1	23.6	0.000
	Female	32.7	28.6	24.5	14.3	18.1	67.4	
Total	Male	60.6	19.6	13.8	6.0	12.4	39.4	0.000
	Female	43.8	25.1	19.9	11.2	16.2	56.1	
Total	Both	53.2	22.0	16.5	8.3	14.1	46.7	

*Less than 20 % of the cells had expected value less than 5

A statistically significant association (*X* ^2^ = 34.405, df = 3, *p* = 0.000) was found between the female gender and the presence and severity of depression (Tables 1 and 2). This trend was present in the four health schools, but it was statistically insignificant in the school of AMS (Table 2). Similarly, a significant association (*X* ^2^ = 34.405, df = 3, *p* = 0.021) was found between the year of study and the presence and severity of depression (Table 1). Among the students of each school alone, the association was statistically insignificant except the school of dentistry (*X* ^2^ = 26.13, df (12), *p* = 0.010), where the highest prevalence was for the fifth and third years (Tables 3 and 4). No significant association was found between students’ marital status or age and the presence or severity of depressive symptoms.

**Table 3:** Association between year of study and level of depressive symptoms among students in each of the four HPs Schools

College of study	Academic year	Minimal *N* (%)	Mild *N* (%)	Moderate *N* (%)	Severe *N* (%)	Total morbidity *N* (%)	Total *N* (%)	*p* value	*X* ^2^ (df)
Medicine	1	56 (55.4)	28 (27.7)	11 (10.9)	6 (5.9)	45 (44.5)	101(100.0)	0.605	10.1 (12)
	2	46 (56.8)	16 (19.8)	12 (14.8)	7 (8.6)	35 (43.2)	81 (100.0)		
	3	42 (48.8)	19 (22.1)	12 (14.0)	13 (15.1)	44 (51.1)	86 (100.0)		
	4	63 (55.8)	20 (17.7)	20 (17.7)	10 (8.8)	50 (44.2)	113 (100.0)		
	5	26 (49.1)	14 (26.4)	7 (13.2)	6 (11.3)	27 (50.9)	53 (100.0)		
	All years	234 (53.8)	97 (22.3)	62 (14.3)	42 (9.7)	201 (46.2)	435		
Dentistry	1	20 (48.8)	9 (22.0)	9 (22.0)	3 (7.3)	21 (51.2)	41 (100.0)	0.010	26.1 (12)
	2	16 (40.0)	8 (20.0)	12 (30.0)	4 (10.0)	24 (60.0)	40 (100.0)		
	3	10 (34.5)	7 (24.1)	10 (34.5)	2 (6.9)	19 (65.5)	29 (100.0)		
	4	33 (76.7)	4 (9.3)	1 (2.3)	5 (11.6)	10 (23.2)	43 (100.0)		
	5	11 (33.3)	9 (27.3)	9 (27.3)	4 (12.1)	22 (66.6)	33 (100.0)		
	All years	90 (48.4)	37(19.9)	41(22.0)	18 (9.7)	96 (51.6)	186		
AMS	1	18 (47.4)	9 (23.7)	11 (28.9)	0 (0.0)	20 (52.6)	38 (100.0)	0.267	11.1 (12)
	2	67 (57.8)	21 (18.1)	19 (16.4)	9 (7.8)	49 (42.2)	116 (100.0)		
	3	61 (51.7)	30 (25.4)	17 (14.4)	10 (8.5)	57 (48.3)	118 (100.0)		
	4	101(54.9)	47 (25.5)	26 (14.1)	10 (5.4)	83 (45.1)	184 (100.0)		
	All years	250 (54.2)	109 (23.6)	73 (15.8)	29 (6.3)	211(45.7)	461		
Nursing	1	0 (0.0)	1 (100.0)	0 (0.0)	0 (0.0)	1 (100.0)	1 (100.0)	0.323	10.3 (12)
	2	10 (83.3)	1 (8.3)	1 (8.3)	0 (0.0)	2 (16.6)	12 (100.0)		
	3	16 (55.2)	4 (13.8)	7 (24.1)	2 (6.9)	13 (44.8)	29 (100.0)		
	4	32 (52.5)	11 (18.0)	11 (18.0)	7 (11.5)	29 (47.5)	57 (100.0)		
	All years	58 (55.8)	18 (17.3)	19 (18.3)	9 (8.7)	46 (44.2)	104

**Table 4:** Association between mean depressive symptoms score versus college and year of study

College	Year	No.	Mean	Std. Deviation	ANOVA *p* value
Medicine	1	101	13.5	8.4	0.405
	2	81	14.4	9.6	
	3	86	16.0	10.5	
	4	113	13.7	10.1	
	5	53	14.7	10.1	
Dentistry	1	41	15.1	9.2	0.000
	2	40	17.1	10.7	
	3	29	17.3	7.6	
	4	43	7.3	12.1	
	5	33	17.7	8.2	
AMS	1	38	14.4	7.1	0.878
	2	116	14.2	8.6	
	3	118	14.1	9.5	
	4	184	13.5	8.2	
Nursing	1	1	14.000		0.160
	2	12	7.1	6.2	
	3	29	13.9	10.3	
	4	61	14.2	10.2	

In the medical and dental schools; the highest prevalence of depressive symptoms was in the third year (51.1 and 65.5 % respectively) and the fifth (50.9 and 66.6 % respectively) years.

## Discussion

The results indicate an alarming outcome particularly among female and dental students. Though the difference was not significant, the prevalence of depressive symptoms was highest among dental followed by medical students and the nursing students having the lowest rate. This finding of non-significant difference with the school is in line with a recent meta-analysis and a qualitative literature review [15, 40, 41].

The high prevalence (47.0 %) in the current study is in line with a recent meta-analysis which found that 49 % of the surveyed medical students have depressive symptoms [42]. It is notable to find in this meta-analysis that the highest prevalence of depression was in the Middle East [15]. However, a study among Polish medical students [43] reported even a higher prevalence rate of depression (56.3 %). The current study outcome is higher than a study among medical students in the University of California using the BDI [44], Quince et al. [45] using the Hospital Anxiety and Depression Scale (HADS-D), and by Seweryn et al. [43] also using the BDI. These studies reported lower rates; i.e. 2.7 to 8.2 % in UK medical students [45], 34.9 % among German medical students and 26 % among Portuguese medical students [43]. The variation of the prevalence rate of depressive symptoms by different studies could be explained in part by the use of different instruments to estimate the rate of depressive symptoms; (e.g. BDI-II versus the CES-D 10 scale). Cultural and economic factors were reported to be associated with the rate of depressive symptoms. Cultural factors such as low sense of control and with less individualistic cultures, and economic factors such as poorer socio-economic population and greater income inequality both were found to play a role [46]. The type of the curriculum was also found to play a role in a study among medical students in Saudi Arabia, which found a statistically significant (*P* = 0.004) higher rate of depressive symptoms among the traditional curriculum students than their system-based counterparts [47].

The negative impact of depression on students continues after graduation [4]. It adversely affects the quality of patient care, patient safety, and professionalism. In other words, its effect extends to the community at large [4, 8]. Therefore, given the high rate of depressive symptoms and the staggering cost, establishing psychological support unit or facility to help students with psychological stresses may be considered.

Even more than the discipline, the gender of the student was found to be the main factor that is associated with depressive symptoms in this study. In line with other research findings [4, 48], the current study showed that the female students on the whole have a higher prevalence of depressive symptoms. A study in Malaysia using the Diagnostic Statistics Manual Four Text Revision (DSM IV-TR) criteria for depression [49] and another study in the United States using the Patient Health Questionnaire-9 [12] that involved pharmacy students have similarly found a higher rate of depressive symptoms among female students compared with their male counterparts. Contrary to these findings, however, a study in a US medical school has reported that male students have more depressive symptoms than the females [50, 51]. The literature is however scarce on this area and additional research on this area could be useful. Age and marital status were not related to this gender difference in the current study.

Other reports support the current study finding that the third year students of the medical and dental schools have higher prevalence of depressive symptoms [10, 52]. These findings have been attributed to the difficulties with the transition from basic science training to clinical training. For some students, interacting with sick people and their suffering and dealing with death are commonly cited stressors associated with anxiety and worries [26]. The finding that the AMS students have a higher prevalence of depressive symptoms in the first year is similar to study’s findings with medical students [53] and university undergraduates [54]. These findings have been mostly attributed to difficulties in adjustment to a new environment, with some students leaving their homes for the first time in their lives, and the high academic demands of the health schools. High exam stress and future employment [52, 55] could be the reasons for the higher prevalence of depressive symptoms in the final year in all courses.

The current study used data on HP students’ depressive symptoms from four schools in one university; using a widely used inventory. Since, most studies have addressed single schools, the results of the study can be used as a proxy indicator of student wellbeing and can be accepted as a baseline for future studies on this area. The stratified proportionate sampling strategy using Microsoft Excel program is another reassuring point for the validity of the study results. However, it is not without limitations including the use of self-administered inventories rather than structured interviews and involving only a single institution. The response rate is acceptable in three schools, it was low among nursing school students due to the continuous exam during the data collection process. Therefore, care was taken to avoid making firm conclusions particularly regarding nursing school results. And finally, the collected questionnaires were not put in sealed envelopes. However, the training session with all the team leaders is likely to combat this drawback.

Future studies should be directed to find out whether the differences in the prevalence of severe depressive symptoms among the students of the different disciplines are due to the innate nature of the discipline or due to the type of curriculum in which they study.

Factors in the curriculum that deserve investigation include self-study time versus classrooms hours per week deserve investigation. At the student level, whether students’ study skills, their resilience or coping skills are associated with the presence of depression needs exploration too. The impact of a Mindfulness and support Program directed to HP students on their Mental Health deserves investigation too [30].

## Conclusion

This study indicates an alarming rate of depressive symptoms among HP students across four schools in the same university; further intervention and clarification research exploring the causes is needed. A higher prevalence of depressive symptoms among female students was found across the four schools. The dental school, the third and fifth years of dental and medical studies, and the first year in AMS studies are mostly associated with depressive symptoms.

## Additional file

Additional file 1: The data of depressive symptoms among Health Professions' students study from King Saud University, Saudi Arabia. (XLSX 159 kb)
